# The impact of blood type on the mortality of patients with severe abdominal trauma: a multicenter observational study

**DOI:** 10.1038/s41598-021-95443-3

**Published:** 2021-08-09

**Authors:** Wataru Takayama, Akira Endo, Kiyoshi Murata, Kota Hoshino, Shiei Kim, Hiroharu Shinozaki, Keisuke Harada, Hiroaki Nagano, Masahiro Hagiwara, Atsuhito Tsuchihashi, Nagato Shimada, Naomi Kitamura, Shunsuke Kuramoto, Yasuhiro Otomo

**Affiliations:** 1 Trauma and Acute Critical Care Center, Tokyo Medical and Dental University Hospital of Medicine, 1-5-45, Yushima, Bunkyo-ku, Tokyo, Japan; 2 Department of Acute Critical Care and Disaster Medicine, Graduate School of Tokyo Medical and Dental University, Tokyo Medical and Dental University, 1-5-45, Yushima, Bunkyo-ku, Tokyo, Japan; 3 The Shock Trauma and Emergency Medical Center, Matsudo City General Hospital, 933-1, Sendabori, Matsudo, Chiba Japan; 4 Department of Emergency and Critical Care Medicine, Fukuoka University Hospital, 7-45-1 Nanakuma, Jonan-ku, Fukuoka, Fukuoka Japan; 5 Department of Emergency and Critical Care Medicine, Nippon Medical School Hospital, 1-1-5, Sendagi, Bunkyo-ku, Tokyo, Japan; 6 Department of Surgery, Saiseikai Utsunomiya Hospital, 911-1, Takebayashi-machi, Utsunomiya, Tochigi Japan; 7 Department of Emergency Medicine, Sapporo Medical University, S1W16, Chuo-ku Sapporo, Hokkaido, Japan; 8 Department of Gastroenterological Breast and Endocrine Surgery, Yamaguchi University Graduate School of Medicine, 1-1-1 Minamikogushi, Ube, Yamaguchi Japan; 9 Department of Surgery, Asahikawa Medical University, 1-1-1 Midorigaoka Higashi 2 Jo, Asahikawa, Hokkaido Japan; 10 Department Surgery, St. Marianna University School of Medicine, 2-16-1, Sugao, Miyamae Ward, Kawasaki, Kanagawa Japan; 11 Department of General Medicine and Emergency Center (Surgery), Toho University Omori Medical Center, 6-11-1 Omorinishi, Ota City, Tokyo, Japan; 12 Department of Surgery, Shiga University of Medical Science Hospital, Seta Tsukinowa-Cho, Otsu, Shiga Japan; 13 Department of Acute Care Surgery, Faculty of Medicine, Shimane University, 89-1, Enya-cho, Izumo, Shimane Japan; 14 Department of Emergency and Disaster Medicine, Tokyo Medical and Dental University, 1-5-45, Yushima, Bunkyo-ku, Tokyo, 113-0034 Japan

**Keywords:** Medical research, Risk factors

## Abstract

Few studies have investigated the relationship between blood type and trauma outcomes according to the type of injury. We conducted a retrospective multicenter observational study in twelve emergency hospitals in Japan. Patients with isolated severe abdominal injury (abbreviated injury scale for the abdomen ≥ 3 and that for other organs < 3) that occurred between 2008 and 2018 were divided into four groups according to blood type. The association between blood type and mortality, ventilator-free days (VFD), and total transfusion volume were evaluated using univariate and multivariate regression models. A total of 920 patients were included, and were divided based on their blood type: O, 288 (31%); A, 345 (38%); B, 186 (20%); and AB, 101 (11%). Patients with type O had a higher in-hospital mortality rate than those of other blood types (22% vs. 13%, *p* < 0.001). This association was observed in multivariate analysis (adjusted odds ratio [95% confidence interval] = 1.48 [1.25–2.26], *p* = 0.012). Furthermore, type O was associated with significantly higher cause-specific mortalities, fewer VFD, and larger transfusion volumes. Blood type O was associated with significantly higher mortality and larger transfusion volumes in patients with isolated severe abdominal trauma.

## Introduction

The ABO blood grouping system of red blood cell (RBC) membrane antigens was devised by Karl Landsteiner at the beginning of the twentieth century^1^. ABH (O) blood group antigens are highly expressed on the surfaces of a variety of human cells and tissues, including platelets, vascular endothelium, and epithelial surfaces^2^, and are known to be the underlying cause of various diseases and clinical conditions. Furthermore, it has been reported that ABO blood type has a profound influence on hemostasis^3–6^. Levels of plasma von Willebrand factor (vWF) and factor VIII (FVIII) are significantly lower in patients with blood type O than in those with non-O blood types; this is associated with an increased risk of bleeding^7^.

Our group previously reported that blood type O was associated with a significantly elevated risk of overall mortality and exsanguination-related death in patients with severe trauma after adjusting for the trauma severity scores and clinical covariates^8^. However, our study was limited by the small number of patients and their heterogeneity, given that a large proportion had severe traumatic brain injury that involves different pathophysiologies in addition to hemorrhage alone.

There have been few published studies analyzing the relationship between blood type and trauma outcomes in the context of specific injury types. In general, injuries to the thoracic and abdominal structures often cause massive bleeding that leads to death. The cause of death due to chest injury is not exclusively hemorrhaging, but could also be a result of hypoxia, cardiac tamponade, air embolisms, and other reasons^9^. However, exsanguination is the leading cause of death in individuals who experience severe abdominal injuries due to blunt^10^ or penetrating trauma^11^. Therefore, patients with such injuries are considered the most suitable for assessing the relationship between blood type and hemorrhaging.

Based on the aforementioned background, the aim of this study was to investigate the outcomes of patients with severe isolated abdominal trauma as influenced by their blood types. We hypothesized that patients with blood type O would experience a higher rate of abdominal trauma-related deaths than would those with other blood types.

## Methods

### Study design and settings

This retrospective multicenter observational study was conducted as a project of the Japanese Society for Abdominal Emergency Medicine. Patient clinical data from 12 emergency hospitals, including the data of some patients in our previous single center study^8^, were collected and analyzed.

The association between ABO blood type and mortality was evaluated in patients with severe isolated abdominal trauma who were transported to a hospital between April 1, 2008 and March 31, 2018. The ethics committees of the Japanese Society for Abdominal Emergency Medicine and of all participating institutes individually approved this study. This study was also approved by the institutional review board of Tokyo Medical and Dental University (#2018-103).

The requirement for individual informed consent was waived because the study was retrospective and relied on anonymized patient and hospital data.

### Study populations

We included consecutive patients with isolated severe abdominal injury, defined as having abbreviated injury scale (AIS)^12^ scores of ≥ 3 for the abdomen and ≤ 2 for other body parts. Patients who met any of the following criteria were excluded from the analysis: (1) those younger than 15 years, (2) those with cardiac arrest upon arrival at the emergency department (ED), (3) those with non-survivable injuries (i.e., abdominal AIS scores of 6), (4) those who were transferred from another hospital, (5) those with a history of taking anti-coagulants or antiplatelet agents, and (6) those whose clinical data were missing or insufficient for analysis.

### Data collection

We collected the patients’ clinical data; these data were collected after excluding the patients who met the exclusion criteria at each hospital. Then, we excluded from the dataset, the data of those patients whose clinical data were missing or insufficient for the analysis. The following information was retrospectively collected from the patients’ medical records at each hospital: age; sex; blood type (A, B, AB, or O); year of injury, type of injury (blunt or penetrating); AIS score of the head and neck, face, chest, abdomen, pelvis and extremities, and surface; injury severity score (ISS); revised trauma score (RTS) calculated based on the Glasgow coma scale; systolic blood pressure; respiratory rate upon arrival at the ED^13^; the total volume of RBCs administered within the first 24 h after ED admission; whether the patient underwent emergency surgery and/or interventional radiology; and survival status upon hospital discharge (i.e., dead or alive). The causes of death were categorized into two groups: exsanguination and other reasons (including multiple organ failure).

### Outcomes

The primary endpoint was in-hospital mortality. The secondary endpoints included cause-specific in-hospital mortality (exsanguination and others), total volume of RBC administered within the first 24 h after arrival at the ED, and ventilator-free days (VFDs)^14^. In Japan, 1 unit of packed RBCs correspond to 120 mL of blood derived from approximately 200 mL of whole blood, while it is approximately 450 mL in the United States. Therefore, we prepared the transfusion unit volumes multiplied by 2.2 to convert them into the United States standard, and have presented them as such herein.

### Statistical analysis

We divided the patients into four groups according to the blood type (i.e., A, B, O, and AB). For univariate analysis, we used Student’s t-test or the Mann–Whitney U-test to compare continuous variables, and we used the χ^2^ test or Fisher’s exact test to compare categorical variables, as appropriate. Categorical variables are reported as numbers (percentages), while continuous variables are reported as medians (interquartile ranges) as appropriate. First, we used a one-way analysis of variance (ANOVA) to assess the differences among the four groups. Second, we performed a post-hoc residual analysis of the primary endpoint to identify the divergent blood type group when a significant difference was observed on one-way ANOVA; the residual was the difference between the observed value and the mean of all the expected values for that group. A significant adjusted standardized residual indicated a significant difference in the corresponding variable. In this analysis, the Bonferroni correction was used to address the issue of multiple comparisons. Third, multivariate analysis was performed using a cluster-weighted generalized linear mixed model to determine whether the blood type was associated with the primary and secondary endpoints. The fixed-effect variables were defined as age, RTS, ISS, while the treating hospital’s unique identifier and the year of injury were incorporated as a random-effect variable. These covariables were selected based on knowledge of the subject matter and the fact that trauma care has been improved over the past decade with better management.

All statistical analyses were performed using the R software (version 3.5.1; R Foundation for Statistical Computing, Vienna, Austria). A command was used to add statistical functions that are frequently used in biostatistics. Two-sided *p*-values < 0.05 were considered statistically significant.

### Ethics approval and consent to participate

The requirement for individual informed consent was waived because the study was retrospective and relied on anonymized patient and hospital data. The ethics committees of the Japanese Society for Abdominal Emergency Medicine and of all participating institutes individually approved this study. This study was also approved by the institutional review board of Tokyo Medical and Dental University (#2018-103). This study complied with the principles of the 1964 Declaration of Helsinki in reviewing and publishing information from the patient’s medical records.

## Results

**Figure 1:**
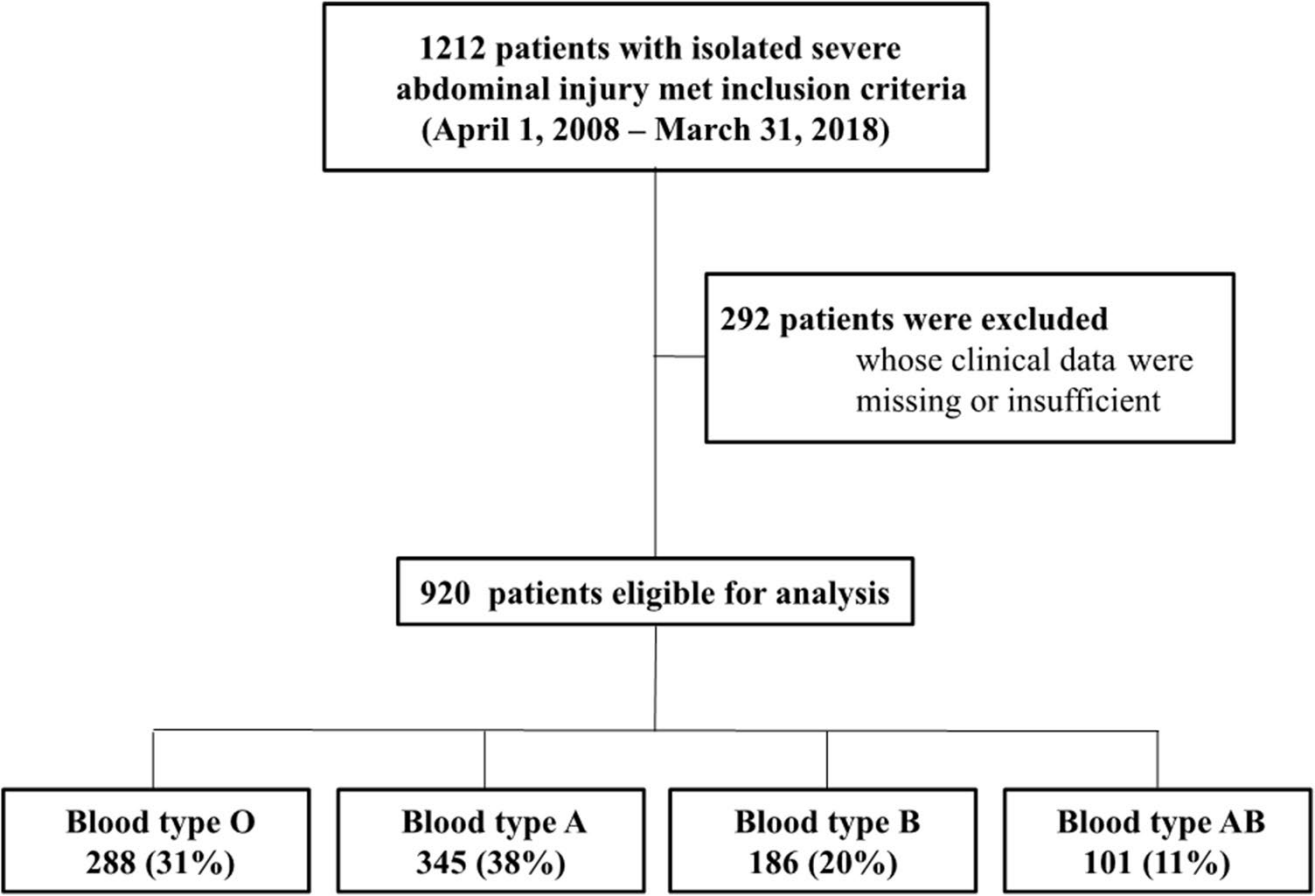
Patient flow diagram.

Of 1212 potentially eligible patients, we analyzed 920 with isolated severe abdominal injury (Fig. 1). The study population was divided into four groups according to the ABO blood type: type O, 288 (31%); type A, 345 (38%); type B, 186 (20%); and type AB, 101 (11%). Table 1 and Supplementary Table S1 present the baseline characteristics of patients in each group. There was no significant difference in the type of injury between groups. The majority of patients sustained blunt trauma and only 9.5% had penetrating injuries (type O, 28 [9.7%]; type A, 40 [11.6%]; type B, 13 [7.0%]; and type AB, 6 [5.9%]). The RTS observed in patients with blood type O was lower than that in patients with other blood types. Furthermore, the number of patients with blood type O who underwent emergency hemostatic intervention was larger than those with other blood types. Table 2 shows patient outcomes according to blood type, in which significant differences were observed throughout. Supplementary Table S2 shows the results of the residual analysis of the primary outcome in each blood group. The adjusted standardized residual of blood type O for the primary outcome was significantly large, indicating a significantly higher association between blood type O and in-hospital mortality compared to other blood types. Similarly, a significantly small adjusted standardized residual for blood type AB indicated a significantly lower association between this blood type and in-hospital mortality compared to other blood types.

**Table 1:** Baseline characteristics of patients categorized according to blood type.

	O, n = 288	A, n = 345	B, n = 186	AB, n = 101	*p*-value
**Characteristics**					
Age (years), median [IQR]	48 [30–68]	42 [29–63]	51 [35–66]	42 [28–66]	0.099
Male sex, n (%)	191 (66.3)	249 (72.2)	133 (71.5)	69 (68.3)	0.410
Blunt trauma, n (%)	260 (90.3)	305 (88.4)	173 (93.0)	95 (94.1)	0.087
RTS, median [IQR]	6.90 [5.70–7.84]	7.84 [6.61–7.84]	7.84 [6.61–7.84]	7.84 [6.81–7.84]	0.007
ISS, median [IQR]	19 [16–25]	18 [14–24]	17 [13–24]	18 [16–24]	0.061
AIS head, median [IQR]	0 [0–1]	0 [0–0]	0 [0–1]	0 [0–1]	0.372
AIS face, median [IQR]	0 [0–0]	0 [0–0]	0 [0–0]	0 [0–0]	0.215
AIS chest, median [IQR]	2 [0–2]	2 [0–2]	1 [0–2]	0 [0–2]	0.083
AIS abdomen, median [IQR]	4 [3, 4]	3 [3, 4]	3 [3, 4]	4 [3, 4]	0.141
AIS pelvis, median [IQR]	1 [0–2]	0 [0–2]	0 [0–2]	0 [0–2]	0.078
AIS surface, median [IQR]	0 [0–1]	0 [0–1]	0 [0–1]	0 [0–1]	0.130
**Interventions**					
Surgery, n (%)	62 (28.5)	45 (13.0)	29 (15.6)	18 (17.8)	< 0.001
IVR, n (%)	40 (13.9)	30 (9.0)	19 (10.2)	12 (11.9)	0.018
Both, n (%)	31 (10.8)	22 (6.4)	15 (8.1)	6 (5.9)	0.018

Categorical variables are expressed as numbers (%), while continuous variables are presented as medians (25th–75th percentiles).

*AIS* abbreviated injury scale, *IQR* interquartile range, *RTS* revised trauma score, *ISS* injury severity score, *IVR* interventional radiology.

**Table 2:** Outcomes of patients categorized by blood type.

Outcomes	O, n = 288	A, n = 345	B, n = 186	AB, n = 101	*p-*value
**Primary outcome**					
In-hospital mortality, n (%)	64 (22.2)	48 (14.0)	27 (14.5)	5 (5.0)	< 0.001
**Secondary outcomes**					
Death due to exsanguination, n (%)	50 (17.3)	40 (11.6)	19 (10.2)	4 (4.0)	< 0.001
Death due to others, n (%)	14 (4.9)	8 (2.3)	8 (4.3)	1 (0.9)	< 0.001
Ventilation-free days, median [IQR]	17 [14–27]	27 [20–28]	26 [17–28]	27 [22–28]	< 0.001
Median RBC volume administered within 24 h, units [IQR]	6 [0–18]	2 [0–10]	0 [0–7]	0 [0–8]	< 0.001

Categorical variables are expressed as numbers (%), continuous variables are presented as medians (25th–75th percentiles).

*IQR* interquartile range, *RBC* red blood cell.

Table 3 showed the results of univariate analyses comparing the characteristics of patients with blood type O to those with other blood types. While there was no significant difference in ISS and AIS for all body regions, patients with blood type O had a significantly lower RTS than did those with other blood types (median [interquartile range {IQR}] = 6.90 [4.45–7.84] vs. 7.84 [6.68–7.84], *p* < 0.001). Table 4 compares the results of univariate analysis of the outcomes of patients with blood type O and those of other blood types. Patients with blood type O had a significantly higher rate of in-hospital mortality than did those with other blood types (22.2% vs. 12.7%, *p* < 0.001), as well as higher rates of death due to exsanguination (17.3% vs. 10.0%, *p* = 0.002), higher rates of death due to other reasons (4.9% vs. 2.7%, *p* = 0.021), and fewer VFDs (median [IQR] = 17 [4–27] days vs. 27 [19–28] days, *p* < 0.001). Furthermore, the volume of administered RBC within 24 h was significantly higher in patients with blood type O than in those with other blood types (median [IQR] = 6 [0–18] units vs*.* 0 [0–10] units, *p* < 0.001).

**Table 3:** Comparison of the characteristics of patients with blood type O versus those with other blood types.

	Type O, n = 288	Non-O type, n = 632	*p*-value
**Characteristics**			
Age, median [IQR]	48 [30–68]	45 [29–65]	0.190
Male, n (%)	191 (66.3)	451 (71.4)	0.141
Blunt trauma, n (%)	260 (90.3)	573 (90.1)	0.324
RTS, median [IQR]	6.90 [4.45–7.84]	7.84 [6.68–7.84]	< 0.001
ISS, median [IQR]	15 [16–24]	14 [14–24]	0.067
AIS head, median [IQR]	0 [0–1]	0 [0–0]	0.121
AIS face, median [IQR]	0 [0–0]	0 [0–0]	0.323
AIS chest, median [IQR]	2 [0–2]	1 [0–2]	0.073
AIS abdomen, median [IQR]	4 [3, 4]	3 [3, 4]	0.290
AIS pelvis, median [IQR]	2 [0–2]	0 [0–2]	0.062
AIS surface, median [IQR]	0 [0–1]	0 [0–1]	0.511
**Interventions**			
Surgery, n (%)	62 (21.5)	92 (14.6)	< 0.001
IVR, n (%)	40 (13.9)	61 (9.7)	< 0.001
Both, n (%)	31 (10.8)	43 (6.8)	< 0.001

Categorical variables are expressed as numbers (%), while continuous variables are presented as medians (25th–75th percentiles).

*RTS* revised trauma score, *ISS* injury severity score, *AIS* abbreviated injury scale, *IQR* interquartile range, *IVR* interventional radiology.

**Table 4:** Comparison of the outcomes of patients with blood type O versus those of other blood types.

	Type O, n = 288	Non-O type, n = 632	*p*-value
**Primary outcome**			
In-hospital mortality, n (%)	64 (22.2)	80 (12.7)	< 0.001
**Secondary outcomes**			
Death due to exsanguination, n (%)	50 (17.3)	63 (10.0)	0.002
Death due to other reasons, n (%)	14 (4.9)	17 (2.7)	0.021
Ventilation-free days, median [IQR]	17 [4–27]	27 [19–28]	< 0.001
Median RBC volume administered within 24 h, units [IQR]	6 [0–18]	0 [0–10]	< 0.001

*IQR* interquartile range, *RBC* red blood cell.

Supplementary Tables S3 and S4 compare the results of univariate analyses of patients with blood type AB and those with other blood types. There were no significant differences between the two groups in terms of ISS and AIS for all body regions, although patients with blood type AB tended to have a higher RTS than did those with other blood types. The blood type AB group had significantly lower in-hospital mortality than the non-AB group (5.0% vs. 17.0%, *p* < 0.001), as well as a lower rate of death due to exsanguination (4.0% vs. 14.0%, *p* < 0.001), a lower rate of death due to other reasons (0.9% vs. 2.9%, *p* = 0.009), and more VFDs (median [IQR] = 27 [22–28] days vs. 25 [11–28] days, *p* < 0.001). The volume of administered RBC within 24 h tended to be lower for patients with blood type AB than for those with other blood types (median [IQR] = 0 [0–8] units vs*.* 4 [0–12] units), although the difference was not significant (*p* = 0.075).

The results of the multivariate analysis of the impact of blood type O on outcomes are shown in Table 5 and in Supplementary Table S5. After controlling for age, ISS, and RTS, blood type O continued to be an independent risk factor for in-hospital mortality (adjusted odds ratio [95% confidence interval {CI} 1.48 [1.25–2.26], *p* = 0.012), death due to exsanguination (adjusted odds ratio [95% CI] 1.86 [1.44–2.46],* p* = 0.010), death due to other reasons (adjusted odds ratio [95% CI] 2.43 [1.34–5.83],* p* = 0.017), fewer VFDs (adjusted differences [95% CI] − 2.31 [− 3.43 to 1.53] days,* p* < 0.001), and the volume of administered RBC within 24 h (adjusted differences [95% CI] = 2.31 [1.94–3.36],* p* < 0.001).

**Table 5:** Multivariate analysis of the impact of blood type O on outcomes using a generalized linear mixed model.

		Adjusted odds ratio [95% CI]		Adjusted difference [95% CI]		*p*-value
**Primary outcome**						
In-hospital mortality		1.48 [1.25–2.26]		–		0.012
**Secondary outcomes**						
Death due to exsanguination		1.86 [1.44–2.46]		–		0.010
Death due to others		2.43 [1.34–5.83]		–		0.017
Median ventilator-free days, days		–		− 2.31 [− 3.43 to − 1.53]		< 0.001
Median RBC volume administered within 24 h, units		–		2.31 [1.94 to 3.36]		< 0.001

*CI* confidence interval, *RBC* red blood cell.

## Discussion

Our analysis of 920 patients with isolated severe abdominal trauma revealed that blood type O is an independent risk factor for in-hospital mortality, death due to exsanguination, a greater volume of RBCs transfused within 24 h, and fewer VFDs. To our knowledge, ours is the first multicenter study to evaluate the impact of blood type on the outcomes of patients with isolated severe abdominal trauma. While a person’s blood type cannot be altered, our data have the potential to be useful not only for predicting outcomes but also as objective indicators of therapeutic strategies.

Damage control strategies for patients with severe abdominal injury generally require a large amount of human and material healthcare resources. As such, identifying patients who require damage control resuscitation is crucial for both patient survival and the efficient utilization of healthcare resources. Our data suggest that blood type may serve as a predictive parameter for determining the level of treatment required for patients with severe abdominal trauma. Notably, although the ISS was similar between two groups, the RTS observed in patients with blood type O was lower than that in patients with other blood type. This difference was speculated to be because the physiological status of patients with blood type O was likely to be deteriorated, due to the exsanguination, even if these patients suffered injuries of the same extent.

Previous studies of trauma patients did not find a correlation between blood type and outcomes^15,16^. There are several potential explanations for this discrepancy. First, since the earlier studies included patients with severe traumatic brain and chest injuries, it is expected that a certain number of patients who died from the causes other than hemorrhage were evaluated. Brain injury is generally the leading cause of death in severe head trauma, and obstructive shock, hypoxia, and air emboli could be one of the causes of death in severe chest trauma. Meanwhile, the cause of early death in most cases of severe abdominal trauma is exsanguination. The present study was designed to examine the more specific association between blood type and exsanguination by limiting target population to severe abdominal trauma. Second, almost all patients analyzed in our study were Japanese, and demographic differences between our study and other may influence the results given that blood type distribution varies by ethnicity and country, and unidentified genetic or environmental factors would modify the blood type-associated risk of bleeding. For example, although the proportion of blood type O in Asians is reported to be approximately 40%, those in African Americans and Hispanics are reported to be 51% and 57%, respectively. In Japan, approximately 99% of the population has a Rh-positive blood type, leaving only 1% Rh negative. Furthermore, a nonsense mutation in the secretor status gene *FUT2*, which controls the expression of ABO antigens in cells and tissues other than RBCs, is differently distributed among ethnic groups^17,18^. Third, compared to studies in other countries^19^, the frequency of penetrating injuries was relatively rare in our cohort. Given the differences in types of injuries and ethnicity-related factors sustained, potentially divergent gene-environment interactions may have influenced the outcomes of various studies. Future investigations of larger global populations are warranted to evaluate the roles of these genetic modifiers.

The association between blood type and coagulation has been explored for over 60 years^20^. The ABH antigenic structures are normally expressed on the N-rinked oligosaccharide chains of vWF, and glycosylation of the H antigen is believed to reduce vWF clearance. Therefore, patients with blood type O, who have unmodified H antigens, have 25–30% lower vWF levels and a decreased activity of FVIII than do those with non-O blood types^21^. vWF plays a decisive role in primary hemostasis by mediating the adhesion of blood platelets to the subendothelium of vessel walls and promotes the aggregation of activated platelets. Several studies have shown that patients with non-O blood type have a potential genetic risk factor for venous thrombosis^6^, alternatively, patients with blood type O with lower plasma vWF levels have a higher risk of hemorrhage^22^. A lower vWF level is a potential explanation for the increase mortality among patients with blood type O, as observed in this study.

It has also been reported that the highest activity of FVIII and vWF is observed in genotype A_2_B, and is high in A_1_A/A_1_B, intermediate in A_1_O/BB/BO, low in A_2_O/A_2_A_2_, and lowest in O/O^23,24^. Although our study was not sufficiently powered to evaluate the least common blood type (i.e., the AB group, which comprised 10% of our study population), this graded genotype effect on FVIII and vWF could explain the higher survival rate of the patients with blood type AB. It has also been reported that the unmodified H antigen is associated with the increased circulation of E-selectin^25–28^, which is expressed on endothelial surfaces in its soluble form and is often correlated with the duration and/or severity of inflammatory conditions^29^.

Trauma can evoke a systemic reaction that includes an acute, nonspecific, immune response that is associated with reduced resistance to infection^30^, and intra-abdominal injury often can cause hyperinflammatory and sepsis status^31^, that could lead to acute respiratory distress syndrome (ARDS). vWF also plays another critical role through immunothrombosis by affecting neutrophil extracellular traps^32^. It has been reported that blood type O is associated with a higher risk of mortality among patients with septic shock^32^ and post burn^33^. Furthermore, although the relationship between differences in blood types and the outcomes of patients with ARDS was reported^34^, the results of association studies are controversial^35^ and we did not evaluate the correlation between ABO blood type and the risk of developing ARDS in this study. While characteristics and mechanisms could explain the increased mortality and the volume of blood transfusion in patients with blood type O as observed in this study, many of the differences in the mechanisms of hemostasis according to blood type remain unclear. Therefore, additional basic and large studies aimed at elucidating the roles of a patient’s blood type in maintaining hemostasis are warranted.

The major strengths of this study were the heterogeneous population derived from 12 different hospitals across Japan, as well as the large number of patients with isolated severe abdominal trauma. The proportions of ABO blood types in our population were consistent with those of published national data in Japan^36^, and our results should therefore be applicable to this country. However, the study also had several limitations that should be considered when interpreting our findings. First, a retrospective design is prone to residual confounding factors and the risk of type I errors, and there was no uniform protocol governing the decision to implement transfusion and/or perform surgery between the treating hospitals. Second, when evaluating outcomes according to the volume of transfusion, patients who died shortly after ED arrival owing to excessive injury were likely to receive small amounts of transfusion, which elicited survival bias. Third, we only evaluated the phenotypes, and not the genotypes, of the ABO blood group or Rh system. Finally, generalizability might be limited because (as mentioned above) almost all members of the study cohort were Japanese. In addition, in this study, the majority of patients sustained blunt trauma. Despite these limitations, we were able to show a significant association between blood type O and outcomes in patients with severe abdominal trauma. Further research beyond this epidemiological study, to evaluate where (through the blood, endothelial cells, FVIII, or vWF) and how (by which mechanism) ABO antigen works, are warranted, to assess the possibility of the novel therapeutic interventions.

## Conclusion

The results of our multicenter retrospective study showed that blood type O is associated with significantly greater mortality in patients with isolated severe abdominal trauma. Further international research to confirm our findings, as well as molecular-level research to investigate the causes of the phenomena we observed, are warranted.

## Supplementary Information

Supplementary Tables.

## Data Availability

The datasets analyzed in this study are not publicly available due to privacy issues, but are available from the corresponding author upon reasonable request.

## Abbreviations

RBC: Red blood cell
vWF: Von Willebrand factor
FVIII: Factor VIII
AIS: Abbreviated injury scale
ED: Emergency department
ISS: Injury severity score
RTS: Revised trauma score
VFDs: Ventilator-free days
ANOVA: Analysis of variance
IQR: Interquartile range
