# Supplementary material for: The impact of blood type on the mortality of patients with severe abdominal trauma: a multicenter observational study
Source: Sci Rep. 2021 Aug 9;11:16147. doi: 10.1038/s41598-021-95443-3 (PMC8352974; doi:10.1038/s41598-021-95443-3)
Supplement: Supplementary file 1 — Supplementary Tables. [file 41598_2021_95443_MOESM1_ESM.docx]

**Title: The impact of blood type on the mortality of patients with severe abdominal trauma: A multicenter observational study**

Wataru Takayama, M.D.^1, 2^; Akira Endo, M.D., Ph.D.^1^ ; Kiyoshi Murata, M.D., Ph.D.^3^; Kota Hoshino, M.D., PhD.^4^; Shiei Kim, M.D., PhD.^5^; Hiroharu Shinozaki, M.D., PhD.^6^; Keisuke Harada, M.D., PhD.^7^; Hiroaki Nagano, M.D., PhD.^8^; Masahiro Hagiwara, M.D., PhD.^9^; Atsuhito Tsuchihashi, M.D., PhD.^10^; Nagato Shimada, M.D., PhD.^11^; Naomi Kitamura, M.D., PhD.^12^; Shunsuke Kuramoto, M.D.^13^ and Yasuhiro Otomo, M.D., Ph.D.^1,2^

| **Supplementary Table S1:** **Comparisons of the number of patients according to year of injury** | | | | | | |
| --- | --- | --- | --- | --- | --- | --- |
|  | | O, n=288 | A, n=345 | B, n=186 | AB, n=101 | **Total, n=920** |
| **Year,** n (%) | |  | | | | |
|  | 2008 | 26 (9.0) | 28 (8.1) | 16 (8.6) | 4 (4.0) | 74 (8.0) |
|  | 2009 | 30 (10.4) | 34 (9.9) | 21 (11.3) | 8 (7.9) | 93 (10.1) |
|  | 2010 | 26 (9.0) | 39 (11.3) | 14 (7.6) | 10 (9.9) | 89 (9.7) |
|  | 2011 | 20 (6.9) | 24 (7.0) | 11 (5.9) | 6 (5.9) | 61 (6.6) |
|  | 2012 | 28 (9.7) | 34 (9.9) | 19 (10.2) | 8 (7.9) | 89 (9.7) |
|  | 2013 | 31 (10.8) | 25 (7.2) | 16 (8.6) | 10 (9.9) | 82 (8.9) |
|  | 2014 | 27 (9.4) | 31 (9.0) | 21 (11.3 | 15 (14.9) | 94 (10.2) |
|  | 2015 | 27 (9.4) | 31 (9.0) | 14 (7.5) | 9 (8.9) | 81 (8.8) |
|  | 2016 | 29 (10.1) | 38 (11.0) | 20 (10.8) | 9 (8.9) | 96 (10.4) |
|  | 2017 | 24 (8.3) | 30 (8.7) | 16 (8.6) | 11 (10.9) | 81 (8.8) |
|  | 2018 | 20 (6.9) | 31 (9.0) | 18 (9.7) | 11 (10.9) | 80 (8.7) |

| **Supplementary Table S2:** **Comparisons of the primary outcome residuals for patients of each blood type** | | |
| --- | --- | --- |
|  | Adjusted standardized residual | Bonferroni-adjusted *p*-value |
| Blood type O | +3.702 | <0.001 |
| Blood type A | −1.125 | n. s |
| Blood type B | −0.477 | n. s |
| Blood type AB | −3.137 | <0.001 |

| **Supplementary Table S3: Characteristics of patients with blood type AB versus those of other blood types** | | | | |
| --- | --- | --- | --- | --- |
| **Characteristics** | | | | |
|  | | **Type AB, n=101** | **Non-AB type, n=819** | ***p*-value** |
|  | Age, median [IQR] | 42 [28–66] | 46 [30–65] | 0.499 |
|  | Male, n (%) | 69 (68.3) | 573 (70.0) | 0.731 |
|  | Blunt trauma, n (%) | 95 (94.1) | 738 (90.1) | 0.124 |
|  | RTS, median [IQR] | 7.84 [6.82–7.84] | 7.84 [6.10–7.84] | 0.066 |
|  | ISS, median [IQR] | 19 [16–24] | 18 [14–24] | 0.392 |
|  | AIS head, median [IQR] | 0 [0–1] | 0 [0–2] | 0.718 |
|  | AIS face, median [IQR] | 0 [0–0] | 0 [0–0] | 0.071 |
|  | AIS chest, median [IQR] | 1 [0–2] | 1 [0–2] | 0.072 |
|  | AIS abdomen, median [IQR] | 4 [3–4] | 3 [3–4] | 0.190 |
|  | AIS pelvis, median [IQR] | 0 [0–2] | 0 [0–2] | 0.117 |
|  | AIS surface, median [IQR] | 0 [0–1] | 0 [0–1] | 0.211 |
| **Interventions** | | | | |
|  | Surgery, n (%) | 18 (17.8) | 136 (16.6) | 0.256 |
|  | IVR, n (%) | 12 (11.9) | 89 (10.9) | 0.144 |
|  | Both, n (%) | 6 (5.9) | 68 (8.3) | 0.029 |
| Categorical variables are expressed as numbers (%), while continuous variables are presented as medians (25^th^–75^th^ percentiles).  Abbreviations: RTS, revised trauma score; ISS, injury severity score; AIS, abbreviated injury scale; IQR, interquartile range; IVR, interventional radiology. | | | | |

| **Supplementary Table S4: Comparison of outcomes between patients with blood type AB and those with other blood types** | | | | |
| --- | --- | --- | --- | --- |
|  | | **Type AB, n=101** | **Non-AB type, n=819** | ***p*-value** |
| **Primary outcome** | | | | |
|  | In-hospital mortality, n (%) | 5 (5.0) | 139 (17.0) | <0.001 |
| **Secondary outcomes** | | | | |
|  | Death due to exsanguination, n (%) | 4 (4.0) | 115 (14.0) | <0.001 |
|  | Death due to others, n (%) | 1 (0.9) | 24 (2.9) | 0.009 |
|  | Ventilation-free days, median [IQR] | 27 [22–28] | 25 [11–28] | <0.001 |
|  | Median volume of RBC administered within 24 h, unit [IQR] | 0 [0–8] | 4 [0–12] | 0.075 |
| Categorical variables are expressed as numbers (%), while continuous variables are presented as medians (25^th^–75^th^ percentiles).  Abbreviations: IQR, interquartile range; RBC, red blood cell. | | | | |

| **Supplementary Table S5: Multivariate analysis of factors influencing patient outcomes and a comparison of explanatory variables** | | | | | | |
| --- | --- | --- | --- | --- | --- | --- |
|  | | | Regression coefficient | Adjusted odds ratio [95% CI] | Adjusted difference  [95% CI] | *p*-value |
| **Primary outcome** | | | | | | |
|  | In-hospital mortality | | | | | |
|  |  | Blood type O | 0.15 | 1.48 [1.25–2.26] | - | 0.012 |
|  |  | Age | 0.02 | 1.03 [1.01–1.04] | - | 0.028 |
|  |  | ISS | 0.24 | 1.21 [1.07–1.34] | - | <0.001 |
|  |  | RTS | −0.50 | 0.58 [0.42–0.67] | - | <0.001 |
| **Secondary outcomes** | | | | | | |
|  | Death due to exsanguination | | | | | |
|  |  | Blood type O | 0.25 | 1.86 [1.44–2.46] | - | 0.010 |
|  |  | Age | 0.02 | 1.03 [0.98–1.13] | - | 0.132 |
|  |  | ISS | 0.07 | 1.07 [1.04–1.12] | - | <0.001 |
|  |  | RTS | −0.61 | 0.69 [0.53–0.77] | - | <0.001 |
|  | Death due to other reasons | | | | | |
|  |  | Blood type O | 0.16 | 2.43 [1.34–5.83] | - | 0.017 |
|  |  | Age | 0.03 | 1.06 [1.01–1.10] | - | 0.034 |
|  |  | ISS | 0.08 | 1.11 [1.01–1.25] | - | 0.005 |
|  |  | RTS | −0.45 | 0.21 [0.12–0.53] | - | <0.001 |
|  | Ventilator-free days | | | | | |
|  |  | Blood type O | −2.31 | - | −2.31 [−3.43 to −1.53] | < 0.001 |
|  |  | Age | −0.21 | - | −0.21 [−0.32 to −0.15] | < 0.001 |
|  |  | ISS | −0.26 | - | −0.26 [−0.5 to −0.12] | < 0.001 |
|  |  | RTS | 2.45 | - | 2.45 [1.95–3.43] | < 0.001 |
|  | Volume of RBCs administered within 24 h | | | | | |
|  |  | Blood type O | 2.31 | - | 2.31 [1.94–3.36] | <0.001 |
|  |  | Age | −0.04 | - | −0.04 [−0.05 to 0.08] | 0.385 |
|  |  | ISS | 0.71 | - | 0.71 [0.35–0.87] | <0.001 |
|  |  | RTS | −1.52 | - | −1.52 [−1.85 to −1.26] | <0.001 |
| Abbreviations: CI, confidence interval; SD, standard deviation; RTS, Revised Trauma Score; ISS, Injury Severity Score; TBI, traumatic brain injury; RBC, red blood cell | | | | | | |
